# Total antioxidant capacity of the diet modulates the association between habitual nitrate intake and cardiovascular events: *A longitudinal follow-up in Tehran Lipid and Glucose Study*

**DOI:** 10.1186/s12986-018-0254-2

**Published:** 2018-02-27

**Authors:** Zahra Bahadoran, Mattias Carlström, Asghar Ghasemi, Parvin Mirmiran, Fereidoun Azizi, Farzad Hadaegh

**Affiliations:** 1 Nutrition and Endocrine Research Center, Research Institute for Endocrine Sciences, Shahid Beheshti University of Medical Sciences, No. 24, Sahid-Erabi St, Yemen St, Chamran Exp, Tehran, 19395-4763 Iran; 2 Department of Physiology and Pharmacology, Karolinska Institutet, Stockholm, Sweden; 3 Endocrine Physiology Research Center, Research Institute for Endocrine Sciences, Shahid Beheshti University of Medical Sciences, Tehran, Iran; 4 Endocrine Research Center, Research Institute for Endocrine Sciences, Shahid Beheshti University of Medical Sciences, Tehran, Iran; 5 Prevention of Metabolic Disorders Research Center, Research Institute for Endocrine Sciences, Shahid Beheshti University of Medical Sciences, Tehran, Iran

**Keywords:** Dietary nitrate and nitrite, Total antioxidant capacity, Cardiovascular disease

## Abstract

**Background:**

Considering the lack of data on the association between habitual dietary intakes of nitrate (NO_3_^−^) and nitrite (NO_2_^−^) and cardiovascular events, we assessed possible effects of dietary NO_3_^−^ and NO_2_^−^, in the context of total antioxidant capacity (TAC) of the diet, with the risk of cardiovascular (CVD) outcomes.

**Methods:**

Adult men and women without CVD (*n* = 2369) were recruited from the Tehran Lipid and Glucose Study and were followed for a mean of 6.7 years. Dietary NO_3_^−^ and NO_2_^−^ intakes, as well as dietary TAC and nitric oxide (NO) index were assessed at baseline (2006–2008). Multivariable-adjusted Cox proportional hazards regression models were used to estimate risk of CVD above and below median of dietary intakes of NO_3_^−^/NO_2_^−^ and dietary TAC and NO index. Due to a significant interaction between NO_3_^−^/NO_2_^−^ intake and TAC, stratified analyses were done for < and ≥ median dietary TAC.

**Results:**

Daily mean (SD) dietary NO_3_^−^ and NO_2_^−^ intakes were 460 (195) and 9.5 (3.9) mg; mean (SD) dietary TAC and NO index was 1406 (740) and 338 (197) μmol trolox equivalent (TE)/100 g. In subjects with lower dietary TAC, higher intake of NO_3_^−^ (≥ 430 mg/d) was accompanied with an increased risk of CVD (HR = 3.28, 95% CI = 1.54–6.99). There were no significant associations between dietary intakes of NO_2_^−^, TAC of the diet and NO index with the occurrence of CVD events during the study follow-up.

**Conclusion:**

High habitual intake of NO_3_^−^, in the context of low TAC of the food, may be associated with the risk of CVD outcomes.

## Background

After the discovery of nitric oxide (NO) production from endogenous reduction of inorganic nitrate (NO_3_^−^) and nitrite (NO_2_^−^), the old perceptions regarding potential hazardous effects of NO_3_^−^ and NO_2_^−^ have changed [1, 2]. Several experimental and clinical studies have been conducted to reveal potential health benefits of these anions in different pathological conditions including hypertension and cardiovascular disease (CVD) [3–5]. Currently, clinical evidence demonstrate that supplementation with NO_3_^−^ and NO_2_^−^, using either inorganic salts or nitrate-rich vegetables (e.g. spinach and beetroot) may promote cardiovascular health [6]. The beneficial effects of NO_3_^−^ and NO_2_^−^ ingestion described in several clinical studies include reduction in blood pressure, measures of arterial stiffness and platelet activity, as well as improvement of vascular function and lipid metabolism [7–9]. Dietary NO_3_^−^ supplementation has also been shown to be able to reverse vascular dysfunction in older adults with moderately increased risk of CVD [10].

Although the health benefits associated with a diet rich in green-leafy vegetables, such as the Mediterranean diet or the Dietary Approaches to Stop Hypertension (DASH) diet in prevention of diabetes and CVD, have been attributed to their high content of NO_3_^−^ [11, 12], there is limited data to confirm longitudinal cardioprotective effects of habitual dietary NO_3_^−^ and NO_2_^−^ intake in epidemiological studies. There are also several issues that need to be clarified in the case of NO_3_^−^ and NO_2_^−^ intake in the general population; e.g. the total amount and the sources of NO_3_^−^ and NO_2_^−^ intakes from the diet, and potential interaction with other dietary components such as antioxidants that may impact on the endogenous metabolic fates of these anions. Despite the beneficial properties of NO_3_^−^/NO_2_^−^ on glucose and insulin homeostasis documented by several pre-clinical and experimental investigations [13], in a recent population-based cohort study we showed that higher NO_2_^−^ intakes from animal-based sources, along with low intake of vitamin C, may increase the risk of type 2 diabetes [14].

Considering the recommendations for advancing knowledge of the relation between dietary NO_3_^−^ and risk of CVD in populations [15], in the current study, we aimed to investigate on the potential effect of dietary NO_3_^−^ and NO_2_^−^ exposure on the development of CVD events during 6.7-year follow-up. It has been suggested that dietary antioxidants could facilitate direct conversion of NO_2_^−^ to NO and enhance NO bioavailability [16]; we therefore hypothesized that the antioxidant capacity of the diet may modify the association between NO_3_^−^/NO_2_^−^ intakes and the risk of CVD. Accordingly, we used interactions term of both NO_3_^−^ and NO_2_^−^ intakes with dietary total antioxidant capacity (TAC) in relation to CVD events. Furthermore, we considered dietary NO index, a novel scoring system combining the inherent NO_3_^−^/NO_2_^−^ content and oxygen radical absorbance capacity (ORAC) score of the food [17].

## Methods

### Study population

**Fig. 1:**
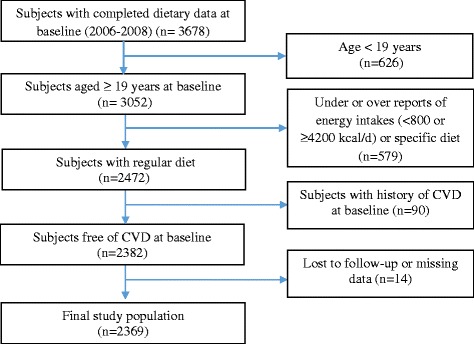
Flowchart for selection of the study participants

This study was conducted within the framework of the Tehran Lipid and Glucose Study (TLGS), an ongoing community-based prospective study being conducted to investigate and prevent non-communicable diseases, in a representative sample in the district 13 of Tehran, the capital city of Iran [18]. During the third phase of the TLGS (2006–2008), a total of 12,523 subjects completed the examinations, of which dietary data from 3678 subjects who agreed to participate and completed the food frequency questionnaire (FFQ) were available [19, 20] . The characteristics of participants who completed the validated FFQ were similar to those of the total population in the third phase of TLGS [21]. For the current analysis, adult men and women (≥ 19 years), free of CVD at baseline, with complete baseline data were included. Participants with under-reported or over-reported energy intake (< 800 kcal/d or > 4200 kcal/d, respectively), or specific diets were excluded. The eligible remaining participants were followed until March 2014, for a mean period of 6.7 years from the baseline examination. Participants who left the study were excluded, and final analyses were conducted on the data of 2369 adults (Fig. 1).

### Demographic, anthropometric and clinical measures

Trained interviewers collected information using standard questionnaires. Detailed measurements of variables in TLGS have been reported elsewhere [18]. Smoking status was obtained using face-to-face interviews; subjects who smoked daily or occasionally were considered current smokers. Weight was measured to the nearest 100 g using digital scales, while the subjects were minimally clothed, without shoes. Height was measured to the nearest 0.5 cm, in a standing position without shoes, using a tape measure. Body mass index (BMI) was calculated as weight (kg) divided by square of the height (m^2^).

For measurements of systolic (SBP) and diastolic blood pressure (DBP), after a 15-min rest in upright position, two measurements of blood pressure were taken on the right arm, using a standardized mercury sphygmomanometer; the mean of the two measurements was considered as the participant’s blood pressure.

### Biochemical measures

Blood samples were taken from all study participants at baseline and at follow-up phases after 12–14 h fasting. Serum glucose was measured by the enzymatic colorimetric method using glucose oxidase. The standard 2-h post-challenge serum glucose (2 h–SG) test was performed using oral administration of 82.5 g glucose monohydrate solution (equivalent to 75 g anhydrous glucose) for all individuals who were not on glucose lowering drugs.

Triglyceride (TG) level was measured by enzymatic colorimetric analysis with glycerol phosphate oxidase. High-density lipoprotein cholesterol (HDL-C) was measured after precipitation of the apolipoprotein B containing lipoproteins with phosphotungstic acid. Analyses were performed using commercial kits (Pars Azmoon Inc., Tehran, Iran) and a Selectra 2 auto-analyzer (Vital Scientific, Spankeren, Netherlands). Both inter- and intra-assay coefficients of variation of all assays were < 5%.

### Habitual dietary assessment

A validated 168-item FFQ was used to assess typical food intakes over the previous year. Trained dietitians, with at least 5 years of experience in the TLGS survey, asked participants to designate their intake frequency for each food item consumed during the past year on a daily, weekly, or monthly basis. Portion sizes of consumed foods reported in household measures were then converted to grams [21]. The validity of the FFQ has been previously evaluated by comparing food groups and nutrient values determined from the questionnaire with values estimated from the average of twelve 24-h dietary recall surveys and the reliability has been assessed by comparing energy and nutrient intakes from two FFQ; Pearson correlation coefficients and intra-class correlation for energy and nutrients showed acceptable agreements between FFQ and twelve 24-h dietary recall surveys, and FFQ1 and FFQ2 [20].

Since the Iranian Food Composition Table is incomplete, and has limited data on nutrient content of raw foods and beverages, to analyze foods and beverages for their energy and nutrient content, the US Department of Agriculture Food Composition Table was used [22].

### Estimation of NO_3_^−^/NO_2_^−^ intake

To estimate dietary intake of NO_3_^−^ and NO_2_^−^, a local database was used [23]. In a recent survey conducted on frequently consumed food items among Iranians, NO_3_^−^ and NO_2_^−^ contents of 87 food items including grains, legumes, fruits and vegetables, dairy products, meats and processed meats were measured. Briefly, a relatively high NO_3_^−^ concentration was observed in breads (~ 50.0 mg 100 g^− 1^). Mean ranges of NO_3_^−^ and NO_2_^−^ in fruits were 7.50–46.8 and 0.15–0.71 mg 100 g^− 1^, respectively. Vegetables with the highest NO_3_^−^ concentrations included radish (626 mg 100 g^− 1^), beetroot (495 mg 100 g^− 1^), tarragon (424 mg 100 g^− 1^), lettuce (365 mg 100 g^− 1^), mint (279 mg 100 g^− 1^), and celery (261 mg 100 g^− 1^). The levels of NO_2_^−^ in vegetables ranged 0.21–0.74 mg 100 g^− 1^. In dairy products, mean NO_3_^−^ and NO_2_^−^ content ranged 0.14–0.45 and 1.26–5.75 mg 100 g^− 1^. Mean NO_3_^−^ and NO_2_^−^ concentrations in meats and processed meats were 5.56–19.4 and 2.93–13.9 mg 100 g^− 1^, respectively [23].

### Validity of NO_3_^−^ and NO_2_^−^ estimation by FFQ

Serum and urinary NO_3_^−^ and NO_2_^−^ concentrations are considered as reliable biomarkers of exogenous exposure to NO_3_^−^ and NO_2_^−^ and the majority of the urinary nitrate can be accounted for by dietary sources [24, 25]. To ensure that estimation of NO_3_^−^/NO_2_^−^ ingestion was associated with empirical measures of exposure [15], in a biomarker study we assessed the correlation between estimated dietary NO_3_^−^ and NO_2_^−^ intake and urinary and serum NO_3_^−^ and NO_2_^−^ levels, in a subsample (*n* = 251) of our cohort population. After adjustment for intra- to inter-individual variance and other potential confounders including age, body mass index and serum creatinine levels, a good agreement was observed between dietary intakes of NO_3_^−^ and NO_2_^−^ and urinary NO_3_^−^ and NO_2_^−^ values (*r* = 0.59, 95% CI = 0.49–0.67, and *r* = 0.29, 95% CI = 0.17–0.41, respectively). The correlations between dietary NO_3_^−^/NO_2_^−^ and serum levels were weak (*r* = 0.19, 95% CI = 0.07, 0.32 and *r* = 0.09, 95% CI = − 0.03, 0.23, respectively).

### Calculation of dietary TAC and NO index

Dietary TAC was estimated based on the ORAC of selected foods reported by Nutrient Data Laboratory of USDA, and expressed as μmol of Trolox Equivalents (TE) per 100 g of foods (μmol TE/100 g) [26]. Dietary NO index was calculated by applying an algorithm considering the NO_3_^−^/NO_2_^−^ concentrations of foods as well as their reported ORAC values: Nitric Oxide Index = [NO_3_^−^ + NO_2_^−^ (mg/100 g) × ORAC (μmol/100 g)] / 1000] [17].

### Definition of terms and outcomes

Details of the collection of CVD outcome data have been described elsewhere [27]. Cardiovascular disease was defined as any coronary heart disease (CHD) events, stroke (a new neurological deficit that lasted ≥24 h), or CVD death (definite fatal myocardial infarction (MI), definite fatal CHD, and definite fatal stroke) [28]. CHD events included cases of definite MI (diagnostic ECG and biomarkers), probable MI (positive ECG findings plus cardiac symptoms or signs plus missing biomarkers or positive ECG findings plus equivocal biomarkers), and angiographic proven CHD. History of CVD was defined as previous ischemic heart disease and/or cerebrovascular accidents. Hypertension (HTN) was defined as SBP ≥ 140 or mm Hg DBP ≥ 90 mmHg, or taking blood pressure lowering medications [29]. Type 2 diabetes (T2D) was defined as fasting serum glucose ≥126 mg/dL or 2 h–SG ≥ 200 mg/dL, or taking anti-diabetic medications [30]. The CVD risk score was calculated according to the sex-specific “general CVD” algorithms and based on age, total cholesterol, HDL-C, SBP, treatment for HTN, smoking, and T2D status [31], which has been validated among Iranian population [32]. This score is the main predictor of CVD events in our population [32].

### Statistical analysis

Dietary intakes were adjusted for total energy intake, according to residuals methods [33]. The incidence of CVD events during the study follow-up was considered as a dichotomous variable (yes/no) in the models.

Median intake of dietary NO_3_^−^ and NO_2_^−^ and NO_3_^−^ + NO_2_^−^ (NOx) was 430, 8.9, and 439 mg/d, respectively. Mean and standard deviation (SD) values and the frequency (%) of baseline characteristics of the study participants were compared above and below median dietary NOx using independent t test or chi square test.

Cox proportional hazards regression models with person-year as the underlying time metric were used to estimate hazards ratios (HRs) and 95% confidence intervals (CIs) for the association between median of dietary NO_3_^−^, NO_2_^−^, NOx, TAC and nitric oxide index and incidence of CVD outcomes. The multivariable-adjusted model was included total energy intakes (kcal/d), dietary total fat (g/d), dietary fiber (g/d), and CVD risk score. Adjustment of CVD risk score, as a continuous potential risk factor of CVD events, improved the stability of our models due to the limited number of events during the study follow-up.

The proportional hazards assumption of the multivariable Cox model was assessed using Schoenfeld’s global test of residuals.

Time to event was defined as time to end of follow-up (censored cases) or time to having an event, whichever occurred first. We censored participants at the time of death due to non-CVD causes, at time of leaving the district, or study follow-up end time of March 2014.

Due to a significant interaction cross-product terms of dietary TAC with both NO_2_^−^ and NO_3_^−^ in the multivariable-adjusted model (*P* < 0.05), stratified analyses were done for dietary NO_2_^−^/NO_3_^−^ and NOx intakes above and below the median dietary TAC (< or ≥1284 μmol TE/ 100 g).

All analyses were performed using IBM SPSS for Windows version 20 and STATA version 12 SE (StataCorp LP, TX, USA), with a two-tailed *P* value< 0.05 being considered significant.

## Results

Mean (SD) age of the study participants was 38.1 (13.3) years and 43.5% were men. Mean (SD) BMI was 26.6 (4.8) kg/m^2^ at baseline. During an average of 6.7 ± 1.4 y of follow-up, 79 participants experienced CVD outcomes. Daily mean (SD) dietary NO_3_^−^ intake in the study population was 460 (195) mg/d, which came almost entirely from plant sources (94.3%); the major contributors to NO_3_^−^ intakes were vegetables (46.1%) and grains (28.8%). Dietary intakes of NO_2_^−^ from animal sources accounted for 42.4% of daily mean intake of NO_2_^−^ and the remainder of NO_2_^−^ intake was derived from plant sources. The major contributors to NO_2_^−^ intake were white rice (17.1%), chicken meat (11.7%), yogurt (6.6%), tomato (5.3%), sausages (4.7%), lamb meat (3.5%), cucumber (3.3%). Mean (SD) dietary TAC and NO index was 1406 (740) μmol TE/100 g and 338 (197).

Baseline characteristics of the cohort population above and below median intake of dietary NOx, are summarized in Table 1. Subjects who had higher intake of NOx were more likely to be older (*P* < 0.05). Mean TAC of diet (1621 vs. 1190 μmol TE/100 g, *P* = 0.001) and dietary NO index (440 vs. 236, *P* = 0.001) was also significantly higher in subjects with higher NOx intakes. There was no significant difference in other baseline characteristics between two groups. Dietary intakes of NO_3_^−^and NO_2_^−^ from vegetables and fruits was significantly higher in subjects who had high-TAC diet (*P* for all < 0.05).

**Table 1:** Baseline characteristics of the study participants (*n* = 2369) above and below median intake of dietary NOx: Tehran Lipid and Glucose Study (2006–2008) to 2014

	*< median NOx*	*≥ median NOx*	*P value*
Age *(y)*	37.4 ± 2.7	38.8 ± 13.8	0.016
Male *(%)*	41.4	45.6	0.022
Smoking *(%)*	12.7	11.3	0.52
Body mass index *(kg/m*^*2*^*)*	26.6 ± 0.1	26.6 ± 0.1	0.92
Systolic blood pressure *(mm Hg)*	109 ± 0.4	110 ± 0.4	0.16
Diastolic blood pressure *(mm Hg)*	72.5 ± 0.3	72.8 ± 0.3	0.38
Fasting serum glucose *(mg/dL)*	88.1 ± 0.5	89.6 ± 0.5	0.32
2 h- serum glucose *(mg/dL)*	97.6 ± 1.0	98.0 ± 1.0	0.79
CVD risk score	19.8 ± 0.72	19.9 ± 0.70	0.66
Diabetes *(%)*	3.4	4.7	0.08
Hypertension *(%)*	8.3	10.3	0.09
Dietary NO_2_^−^ *(mg/d)*	8.7 ± 3.9	10.3 ± 3.7	0.001
From vegetables *(mg/d)*	0.65 ± 0.43	1.1 ± 0.69	0.01
From fruits *(mg/d)*	0.87 ± 0.56	2.4 ± 1.4	0.001
From grains *(mg/d)*	2.1 ± 1.3	2.0 ± 1.1	0.23
From legumes *(mg/d)*	0.07 ± 0.09	0.12 ± 0.16	0.11
From dairy *(mg/d)*	8.7 ± 5.5	17.5 ± 10.1	0.001
From meats *(mg/d)*	5.2 ± 4.2	6.6 ± 4.9	0.001
From processed meats *(mg/d)*	1.5 ± 3.0	1.6 ± 2.1	0.78
Dietary NO_3_^−^ *(mg/d)*	359 ± 119	562 ± 203	0.001
From vegetables *(mg/d)*	158 ± 99.0	261 ± 162	0.001
From fruits *(mg/d)*	35.2 ± 22.8	94.5 ± 56.4	0.001
From grains *(mg/d)*	136 ± 79.01	133 ± 69.0	0.24
From legumes *(mg/d)*	2.7 ± 3.5	3.9 ± 5.4	0.08
From dairy *(mg/d)*	0.8 ± 0.4	1.5 ± 0.8	0.001
From meats *(mg/d)*	1.9 ± 1.7	2.4 ± 1.7	0.001
From processed meats *(mg/d)*	0.8 ± 2.05	0.8 ± 1.2	0.81
Dietary TAC *(μmol TE/100 g)*	1190 ± 632	1621 ± 777	0.001
Nitric oxide index	236 ± 107	440 ± 213	0.001

Data are mean ± SD unless stated otherwise (independent t-test and chi-square test were used for continuous and dichotomous variable, respectively)

Median intake of dietary nitrate, nitrite and nitrate+nitrite was 430, 8.9, and 439 mg/d, respectively

NO_3_^−^, nitrate, NO_2_^−^, nitrite; NOx, NO_3_^−^ + NO_2_^−^; *CVD* cardiovascular disease, *TAC* total antioxidant capacity

The association of dietary NO_3_^−^, NO_2_^−^ and NOx intakes with the incidence of CVD events, above and below median TAC of diet, are reported in Table 2. In subjects with lower dietary TAC, higher intake of NO_3_^−^ was accompanied with an increased risk of CVD (HR = 3.28, 95% CI = 1.45–6.99). There were no significant association between dietary intakes of NO_2_^−^ with the occurrence of CVD events during the study follow-up.

**Table 2:** The association of dietary nitrate, nitrite and NOx intakes with the incidence of CVD events after 6.7 years of follow-up: Tehran Lipid and Glucose Study 2006–2008 to 2014 (n = 2369)

	NO_3_^−^ (≥ 430 mg/d)	NO_2_^−^ (≥ 8.9 mg/d)	NOx (≥ 439 mg/d)
< Median dietary TAC	21/332 ^a^	10/309	21/443
*Crude*	2.89 (1.55–5.37)	0.95 (0.46–1.95)	1.21 (0.64–2.25)
*Adjusted* ^*b*^	3.28 (1.54–6.99)	0.64 (0.25–1.62)	0.91 (0.41–1.96)
≥ Median dietary TAC	30/853	32/876	31/742
*Crude*	1.25 (0.59–2.64)	1.56 (0.69–3.54)	1.42 (0.75–2.70)
*Adjusted*^*1*^	1.10 (0.46–2.61)	2.14 (0.84–5.45)	1.19 (0.54–2.62)

Hazards ratio (95% CI); Cox proportional hazards regression models were used

NO_3_^−^, NO_2_^−^, and NOx were included as dichotomous variables (< and ≥ median intakes). Median intake of dietary nitrate, nitrite and nitrate+nitrite was 430, 8.9, and 439 mg/d, respectively

Median dietary TAC was 1284 μmol TE/100 g

*TAC* total antioxidant capacity; NO_3_^−^, Nitrate; NO_2_^−^, Nitrite; NOx, NO_3_^−^ + NO_2_^−^

^a^ n (event)/N (total)

^b^ Adjusted for dietary total energy intakes (kcal/d), total fat (g/d), dietary fiber (g/d) and cardiovascular disease risk score

The associations of dietary TAC and NO index with the incidence of CVD after 6.7 years of follow-up are reported in Table 3. After adjustment of all potential confounders, there were no significant associations between TAC of the diet (HR = 0.92, 95% CI = 0.59–1.59) and dietary NO index (HR = 1.12, 95% CI = 0.69–1.83) with the occurrence of CVD events during the study follow-up.

**Table 3:** The association of dietary TAC and NO index with the incidence of CVD after 6.7 years of follow-up: Tehran Lipid and Glucose Study 2006–2008 to 2014 (*n* = 2369)

	TAC (≥ 1284 μmol TE/100 g)	Nitric oxide index (≥ 300)
n/N ^a^	39/1176	42/1179
Crude	0.99 (0.63–1.54)	1.13 (0.73–1.77)
Adjusted ^b^	0.97 (0.59–1.59)	1.12 (0.69–1.83)

Hazards ratio (95% CI); Cox proportional hazard regression models were used

TAC and nitric oxide index were included as dichotomous variables (< or ≥ median intakes)

Median of dietary TAC and nitric oxide index was 1284 μmol TE/100 g and 300, respectively

*TAC* total antioxidant capacity, *CVD* cardiovascular disease, *TE* trolox equivalent; NOx, NO_3_^−^ + NO_2_^−^

^a^ n (event)/N (total)

^b^ Adjusted for dietary total energy intakes (kcal/d), total fat (g/d), dietary fiber (g/d) and cardiovascular disease risk score

## Discussion

In this longitudinal population-based study, we are the first to demonstrate the potential impact of habitual dietary NO_3_^−^ and NO_2_^−^ intake along with dietary antioxidant capacity on the risk of CVD. Independent of other risk factors, higher intake of dietary NO_3_^−^ was significantly associated with an increased risk of CVD in subjects consuming food with low antioxidant capacity, dietary NO_2_^−^ intake was not related to incidence of CVD after a median 6.7 years of follow-up. We also assessed probable association of NO index (i.e. combines inherent NO_3_^−^/NO_2_^−^ content and ORAC score of the food), with the development of CVD events; by using this novel scoring system we did not observed any significant association between dietary NO index and the incidence of CVD events. Recently, it was shown that serum NO_3_^−^/NO_2_^−^ are independent risk factors for the development of CVD among a Tehranian adult population [34]; in the current study, we extended our previous work by showing the positive association of dietary NO_3_^−^ with development of CVD in subjects who had a low-TAC diet.

Although accumulating evidence supports biological cardioprotective effects of inorganic NO_3_^−^/NO_2_^−^ following a short-time supplementation, in both experimental and clinical studies, [3, 35, 36], cardiovascular responses to habitual dietary intake of NO_3_^−^/NO_2_^−^ in a long-term period still remains unknown and seems to be a challenging issue [37]. Lack of improvement in blood pressure or atherogenesis as well as dropped levels of serum NO_2_^−^ during a prolonged inorganic NO_3_^−^ supplementation, has been recently showed in low density lipoprotein receptor knockout mice [38], suggests a possible negative feedback mechanism in long-term consumption of inorganic NO_3_^−^ which seems to rebalance the NO_3_^−^-NO_2_^−^-NO pathway [37].

Our findings in the current study also reinforce possible importance of antioxidant compounds in modification of cardiovascular outcomes in longitudinal exposure of dietary NO_3_^−^. In agreement with our idea, Blekkenhorst et al. in a 15-years follow-up of older adults reported that NO_3_^−^ intake from vegetables, as a high-TAC food group, was inversely associated with atherosclerotic vascular disease (ASVD) mortality independent of lifestyle and CVD risk factors; whereas, non-vegetable NO_3_^−^ intake was not related to ASVD (HR = 1.03, 95% CI = 0.85–1.25), *P* = 0.754) [39]. The usual dietary consumption of NO_3_^−^ and NO_2_^−^ in our study was approximately twofold of the acceptable daily intake (ADI) values and higher than the other population. In a recent experimental study we demonstrated that long-term dietary supplementation with high dose of inorganic NO_3_^−^ was associated with down-regulation of endothelial NO synthase function and elevated blood pressure [40]. The existence of similar crosstalk between the NO_3_^−^-NO_2_^−^-NO pathway and the NO synthase pathway was not assessed in the present study, but may contribute to the increased CVD risk in the population consuming food with high NO_3_^−^ and low TAC. Our observations may indicate that beneficial effects of NO_3_^−^/NO_2_^−^ can be expected in its acceptable range of intakes and from the specific food sources. It should be noted that high intake of NO_3_^−^ with low TAC was related to high intake of grains, meats and processed meats along with low consumption of fruits and vegetables. Increased risk of CVD events in participants with higher NO_3_^−^/NO_2_^−^ along with a low-TAC diet, therefore may be attributed to higher intake of meats and processed meats, regardless of dietary NO_3_^−^/NO_2_^−^ intakes.

Difference between short-term effects of rich NO_3_^−^/NO_2_^−^ extracts compared to its regular intake in a long-term period, in the context of diverse dietary patterns may be another explanation for our findings. In our previous studies, we also observed that higher intakes of NO_2_^−^, especially from animal sources, was accompanied with an elevated incidence of type 2 diabetes in subjects who consumed a lower vitamin C [14]. It seems that antioxidant components of our daily diet could modulate endogenous pathways of NO_3_^−^/NO_2_^−^ metabolism and modify the risk-benefit outcomes of these anions in the body [41, 42].

Our study had some strengths and limitations. This study was conducted in the framework of a prospective population-based design, with a high rate of follow-up completeness. We used a validated comprehensive FFQ to assess regular dietary intakes of the participants, and estimated NO_3_^−^/NO_2_^−^ exposure based on a national database of NO_3_^−^/NO_2_^−^ content [23]. Use of validated CVD risk score in the multivariable-adjusted models allowed us to account for major CVD confounders without adding many variables that would lead to instability of our models.

Use of ORAC values to calculate NO index is a major limitation of this study since the values of antioxidant capacity have no relevance to the effects of specific bioactive compounds in foods; in addition, ORAC assay using different substrates do not provide comparable results. Based on these evidence, USDA’s Nutrient Data Laboratory removed the ORAC Database for selected foods from the website in 2012 [43]. Lack of data on income level, alcohol consumption, and dietary supplement use, as potential moderator of CVD risk, and also lack of data on serum TAC and inflammatory markers should be considered as limitations of this analysis. Lack of data on dietary supplements could also affect our estimation of dietary TAC. Another main limitation in this study was a lack of information regarding water intake and NO_3_^−^/NO_2_^−^ concentration of drinking water, at baseline. However, our recent estimation of intake from drinking water in a subsample of TLGS population, showed a relatively low contribution of drinking water in overall NO_3_^−^/NO_2_^−^ exposure compared to its dietary sources, in our population (6.7% and 26.6% for NO_3_^−^ and NO_2_^−^, respectively) [14]. Due to potential changes in an individual’s diet and NO_3_^−^/NO_2_^−^ content of food items, as well as changes in other CVD risk factors during the study follow-up, some degree of misclassification might have occurred which could lead to biased estimated hazards ratios towards the null, as inherent in any prospective study.

It should be noted that our findings may not be generalized to other populations due to different NO_3_^−^/NO_2_^−^ content of the food items especially vegetables and different dietary patterns and food habits among different populations.

## Conclusion

The results of our investigation suggest that high habitual intake of NO_3_^−^ in the context of low TAC diet may be associated with CVD events, independent of other known risk factors. A null association of dietary NO index, which is calculated from NO_3_^−^ and NO_2_^−^ content and ORAC score of the diet, also indicates that the suggested adverse effect of NO_3_^−^ may be modified by the antioxidant capacity of the foods.

## Abbreviations

2 h–SG: 2-h serum glucose
ASVD: Atherosclerotic vascular disease
BMI: Body mass index
CHD: Coronary heart disease
CVD: Cardiovascular disease
DASH: Dietary approach to stop hypertension
DBP: Diastolic blood pressure
FFQ: Food frequency questionnaire
HDL-C: High-density lipoprotein cholesterol
HR: Hazards ratio
HTN: Hypertension
NO: Nitric oxide
NO_2_^-^: Nitrite
NO_3_^-^: Nitrate
ORAC: Oxygen radical absorbance capacity
SBP: Systolic blood pressure
SD: Standard deviation
TAC: Total antioxidant capacity
TG: Triglycerides
TLGS: Tehran Lipid and Glucose Study
